# Bovine leukemia virus long terminal repeat variability: identification of single nucleotide polymorphisms in regulatory sequences

**DOI:** 10.1186/s12985-018-1062-z

**Published:** 2018-10-25

**Authors:** Aneta Pluta, Marzena Rola-Łuszczak, Renée N Douville, Jacek Kuźmak

**Affiliations:** 1 Department of Biochemistry, National Veterinary Research Institute, Puławy, Poland; 2 Department of Biology, The University of Winnipeg, Winnipeg, MB Canada; 3 Department of Immunology, University of Manitoba, Winnipeg, MB Canada

**Keywords:** Bovine leukemia virus, Long-terminal repeat (LTR), Virus variability, Transcription factors

## Abstract

**Background:**

Limited data are available on the incidence of variations in nucleotide sequences of long terminal repeat (LTR) regions of Bovine Leukemia Virus (BLV). Consequently, the possible impact of SNPs on BLV LTR function are poorly elucidated. Thus, a detailed and representative study of full-length LTR sequences obtained from sixty-four BLV isolates from different geographical regions of Poland, Moldova, Croatia, Ukraine and Russia were analyzed for their genetic variability.

**Methods:**

Overlap extension PCR, sequencing and Bayesian phylogenetic reconstruction of LTR sequences were performed. These analyses were followed by detailed sequence comparison, estimation of genetic heterogeneity and identification of transcription factor binding site (TFBS) modifications.

**Results:**

Phylogenetic analysis of curated LTR sequences and those available in the GenBank database reflected the acknowledged *env* gene classification of BLV into 10 genotypes, and further clustered analysed sequences into three genotypes - G4, G7 and G8. Additional molecular studies revealed the presence of 97 point mutations distributed at 89 positions throughout all 64 LTR sequences. The highest rate of variability was noted in U3 and U5 subregions. However, the variability in regulatory sequences (V_R_) was assessed as lower than the variability within non-regulatory sequences (V_NR_) for both, U3 and U5 subregions. In contrast, V_R_ value for R subregion, as well as for the total LTR, was higher than the V_NR_ suggesting the existence of positive selection. Twelve unique SNPs for these LTR sequences localized in regulatory and non-regulatory elements were identified. The presence of different types of substitutions lead to the abrogation of present or to the creation of additional TFBS.

**Conclusion:**

This study represents the largest study of LTR genetic variability of BLV field isolates from Eastern part of Europe. Phylogenetic analysis of LTRs supports the clustering BLV variants based on their geographic origin. The SNP screening showed variations modifying LTR regulatory sequences, as well as altering TFBS. These features warrant further exploration as they could be related to proviral load and distinctive regulation of BLV transcription and replication.

**Electronic supplementary material:**

The online version of this article (10.1186/s12985-018-1062-z) contains supplementary material, which is available to authorized users.

## Background

Bovine leukemia virus (BLV) is the etiological agent of enzootic bovine leucosis (EBL), a chronic, lymphoproliferative disease associated with persistent lymphocytosis and B-cell lymphomas. BLV, together with human T-cell leukemia viruses type 1 and 2 (HTLV-1, HTLV-2), belong to the genus *Deltaretrovirus* of the family *Retroviridae*. BLV virions contain a diploid RNA genome that is reverse transcribed into double-stranded DNA, which is ultimately integrated into the host genome in the form of a provirus. The BLV genome consists of *gag, pol and env*, structural genes and a region X which contains several open reading frames for Tax, Rex, R3 and G4 regulatory proteins [1]. The Tax protein is involved in activation of transcription of viral mRNA, while Rex protein regulates the synthesis of BLV structural proteins [2, 3].

Whole BLV genome is flanked at both 5′ and 3′ ends by long terminal repeat (LTR) sequences [4, 5]. Each LTR is composed of three regions called U3, R and U5. U3 plays a crucial role in the induction of BLV transcription, since its contains the viral promoter and several regulatory elements critical for the modulation of promoter activity [6–9]. The major regulatory elements are three repeated 21-bp Tax-responsive elements (TRE) that include imperfectly conserved cyclic AMP-responsive elements (CRE) matching the consensus sequence 5′-TGACGTCA-3′. CRE sites are used by cellular transcription factors ATF-1 and ATF-2, as well as cyclic AMP response element binding (CREB) proteins [10]. The virus-encoded Tax transactivator increases the DNA binding activity of CREB/ATF proteins by interacting with their bZip domains, which positively regulates the activation of BLV transcription. Besides the CRE elements, each TRE sequence includes an E box as a target sequence for the AP4 transcription factor. Additionally, the U3 region contains several other response elements, such as: a nuclear factor-κB (NF-κB) binding site, which permit activation of transcription in presence of NF-κB p50 and p65 proteins, a glucocorticoid response element (GRE) conferring responsiveness to dexamethasone in the presence of the Tax, and PU.1/Spi-B binding sites for ETS transcription factor family proteins [7–9]. Other elements which were involved in regulation of virus transcription are located downstream of transcription initiation site in R-U5 regions and include an upstream stimulatory factor (USF) binding site, downstream activator sequence (DAS) and an interferon regulatory factor (IRF) binding site, respectively [11–13].

Several studies have demonstrated that sequence variations in retroviral LTRs may affect interactions with transcription factors, leading to altered expression of viral genes and ultimately virus replication. Indeed, it was shown that mutations in lentiviral LTRs caused distinct transcriptional activities of HIV-1 and small ruminant lentiviruses [14, 15]. Genetic diversity within LTRs was also associated with decreased pathobiology in lungs or increased neurovirulence in sheep infected with ovine lentivirus [16, 17]. A correlation was also found between mutations within the Tax-responsive element of human HTLV-1 and increased virus replication [18]. Together, this points to subtle differences in transcription factor biding sites potentially impacting the global regulation and clinical outcomes of retrovirus infection.

Analysis of BLV LTR sequences is limited to few studies; however, the most extensive nucleotide analysis focused on the *env* gene, with the identification of ten distinct genotypes thus far [19–21]. Significant variability within 5′ LTR sequences were found in BLV isolates from North America, Costa Rica, Japan and Belgium, and grouped in 7 genetic groups. Notably, these LTR sequences were more conserved than non-regulatory regions [22]. Recently, phylogenetic analysis of 5′ LTR sequences of BLV isolates from Brazil showed that these sequences clustered into five well-defined groups, with accumulation of nucleotide variability in the R-U5 region [23]. LTR variability has been correlated with altered BLV virulence and pathogeny [24]. In addition, nucleotides polymorphisms found within the LTR region was associated with the emergence of two distinct infection profiles [25].

Sero-epidemiological surveys revealed that BLV infection is widely disseminated throughout the world, with a high prevalence in North and South America, as well as some Asiatic and Middle Eastern countries [26]. Poland has declared freedom from EBL in 2017; however, the infection is still present in East European countries like Ukraine, Moldova and Russia. A common economic zone with these countries still creates a potential for negative impact on the protection of the cattle populations and maintenance of BLV-free herd status in Poland. EBL risks in dairy herds must be considered given the development of trade with Eastern European countries.

In this study, full-length LTR sequences obtained from 64 BLV isolates from different geographical regions of Poland, Moldova, Croatia, Ukraine and Russia were analyzed for their genetic variability. We have found new SNPs modifying LTR regulatory sequences and provided further evidence for positive selection pressure in particular regions of the LTR.

## Methods

### Sample collection

Blood samples were collected from 64 cattle, serologically positive for BLV infection, as was diagnosed by different commercially available ELISA kits (IDEXX, blocking ELISA) or AGID (agar gel immunodiffusion) tests. The animals came from 47 herds, located in 19 geographically distinct regions in five countries: Poland, Moldova, Croatia, Ukraine and Russia. Available information about the isolates is summarized in Table 1. Blood samples from Russia, Moldova and Ukraine were originally obtained from collaborating laboratories: Urals State Scientific Research Institute of Veterinary Medicine, Russia; Republican Center for Veterinary Diagnostic, Moldova and National Scientific Center Institute of Experimental and Clinical Veterinary Medicine, Ukraine and were sent to the National Veterinary Research Institute in Pulawy in the form of dry pellets of peripheral blood leukocytes (PBLs). DNA sample from two cattle from Croatia was kindly supplied by Dr. D. Balic (Veterinary Institute Vinkovci, Croatia). Forty-seven blood samples from Poland were selected by local diagnostic laboratories during EBL monitoring programme between 2013 and 2016.

**Table 1:** Identity and origin of the sequences analysed in the study

GenBank accession no	Collection date	Geographic origin & voivodeship	Genotype	Identity code & source
MH423660	2013	Poland: Kuyavian-Pomeranian	4-III	0221AGD_K-P, this work
MH423661		Poland: Kuyavian-Pomeranian	4-III	0222GD_K_P, this work
MH407744		Poland: Silesian	4-II	020B_S, this work
MH423662		Poland: Lower Silesian	8-I	0139O_L_S, this work
MH423663		Poland: Lower Silesian	8-I	0135O_L_S, this work
MH423664		Poland: Lower Silesian	8-I	0133O_L_S, this work
MH423665		Poland: Lower Silesian	8-I	0132O_L_S, this work
MH423666		Poland: Podlaskie	8-I	019WM_P, this work
MH423667		Poland: Podlaskie	7-I	0184S_P, this work
MH423668		Poland: Warmian-Masurian	4-I	030O_W-M, this work
MH423669		Poland: Warmian-Masurian	4-III	3208M_W-M, this work
MH423670		Poland: Warmian-Masurian	4-III	3206M_W-M, this work
MH423653	2014	Poland: Warmian-Masurian	8-I	0405W_W-M, this work
MH423654		Poland: Warmian-Masurian	4-I	4W_W-M, this work
MH423655		Poland: Warmian-Masurian	4-I	0071B_W-M, this work
MH423656		Poland: Warmian-Masurian	4-I	0072B_W-M, this work
MH407735		Poland: Warmian-Masurian	4-II	0253G_W-M, this work
MH407736		Poland: Warmian-Masurian	4-II	0252G_W-M, this work
MH748226		Poland: Podlaskie	7-II	03511M_P, this work
MH423657		Poland: Warmian-Masurian	4-II	0242K_W-M, this work
MH423658		Poland: Podlaskie	7-I	03513M_P, this work
MH407742		Poland: Silesian	4-II	0741M_S, this work
MH407743		Poland: Silesian	4-II	053K_S, this work
MH423659		Poland: Lodz	4-III	047P_Lodz, this work
MH423647	2015	Poland: Warmian-Masurian	4-I	0094B_W-M, this work
MH423648		Poland: Warmian-Masurian	4-I	0101B_W-M, this work
MH423649		Poland: Warmian-Masurian	4-I	0102B_W-M, this work
MH423650		Poland: Warmian-Masurian	4-I	017B_W-M, this work
MH423651		Poland: Podlaskie	8-I	0081Z_P, this work
MH407740		Poland: Silesian	4-II	0131Z_S, this work
MH407741		Poland: Silesian	4-II	026Z_S, this work
MH423652		Poland: Lublin	8-I	011TL_L, this work
MH407738	2016	Poland: Podlaskie	4-I	035S_P, this work
MH423633		Poland: Greater Poland	4-I	015P_G_P, this work
MH423634		Poland: Podlaskie	4-I	0168BP_P, this work
MH423635		Poland: Podlaskie	4-I	0167BP_P, this work
MH423636		Poland: Podlaskie	4-I	0166BP_P, this work
MH423637		Poland: Podlaskie	4-I	01610BP_P, this work
MH423638		Poland: Warmian-Masurian	7-I	019W_W-M, this work
MH423639		Poland: Warmian-Masurian	8-I	010W_W-M, this work
MH423640		Poland: Warmian-Masurian	4-I	038W_W-M, this work
MH423641		Poland: Warmian-Masurian	4-III	0371B_W-M, this work
MH423642		Poland: Warmian-Masurian	4-III	0374B_W-M, this work
MH423643		Poland: Warmian-Masurian	4-III	0378B_W-M, this work
MH423644		Poland: Warmian-Masurian	4-I	009B_W-M, this work
MH423645		Poland: Warmian-Masurian	4-I	006B_W-M, this work
MH423646		Poland: Kuyavian-Pomeranian	4-I	014NN_K-P, this work
MG407617	2012	Moldova: Region Hincesti	7-II	13MD, this work
MG407616		Moldova: Region Hincesti	7-II	16MD, this work
MG407618		Moldova: Region Riscani	7-II	1MD, this work
MG407619		Moldova: Region Riscani	4-III	8MD, this work
MH423631	2009	Croatia	8-II	VRA_Cro, this work
MH423630		Croatia	8-II	ORA_Cro, this work
MH423632		Ukraine: Rivnenska Oblast	4-III	42UA_Ukr, this work
MH423671		Russia: Kurgan area	4-IV	3K_Rus, this work
MH423672		Russia: Chyelabinsk	4-III	4Z_Rus, this work
MH423673		Russia: Krasnodar Territory	4-III	5.RU_Rus, this work
MH423674		Russia: Tyumen	4-IV	6T_Rus, this work
MH423675		Russia: Chyelabinsk	4-III	8Ch_Rus, this work
MH407739		Poland: West Pomeranian	7-I	146_W_P, this work
MH407737		Poland: Opole	4-II	301_O, this work
MH423676		Poland: Greater Poland	4-III	58_G_P, this work
MH423677		Poland: Greater Poland	4	68_G_P, this work
MH423678		Poland: Masovian	4	297WS_M, this work
AB934282.1	2014	Japan	1	Mekata et al. (2014)^b^
EF600696.1	1985	USA, subclone pBLV913	1	Derse, 1985 [42]
HE967301.1	2012	Uruguay	1	Moratorio et al. (2012)^a^
LC164084.1	2016	Japan	1	Murakami, 2016 [43]
AF257515.1	2000	Argentina	2	Dube, 2000 [44]
LC080655.1	2007	Paraguay	2	Polat, 2016 [19]
LC080654.1	2008	Peru	2	Polat, 2016 [19]
LC080662.1	2008	Bolivia	9	Polat, 2016 [19]
LC080666.1	2008	Bolivia	9	Polat, 2016 [19]
DQ288214.1	2005	Japan	3	Zhao, 2007 [22]
DQ288182.1	2005	USA	3	Zhao, 2007 [22]
DQ288222.1	2005	Costa-Rica	5	Zhao, 2007 [22]
DQ288228.1	2005	Costa-Rica	5	Zhao, 2007 [22]
LC080657.1	2007	Paraguay	6	Polat, 2016 [19]
LC080658.1	2007	Paraguay	6	Polat, 2016 [19]
LC154848.1	2014	Myanmar	10	Polat, 2017 [45]
LC154849.1	2014	Myanmar	10	Polat, 2017 [45]

^a^Moratorio et al. (2012) unpublished, direct submission to GenBank

^b^Mekata et al. 2014, unpublished, presented and classified by Polat et al. 2017 [45]

### DNA isolation

PBLs were isolated from 10 mL of blood by centrifugation at 1500 g for 25 min. Erythrocytes were haemolysed by osmotic shock with H_2_O and 4.5% NaCl. Afterwards, the cells were washed twice with PBS, aliquoted (5 × 10^6^ cells), and stored as pellet at − 80 °C until DNA extraction. Five archived samples collected in 2009 from BLV positive cows from Poland were stored as DNA samples at − 20 °C until further use. Genomic DNA was extracted using a DNeasy Blood & Tissue Kit (Qiagen), following the manufacturer’s instructions. For each sample, genomic DNA concentration was measured using a nanophotometer (IMPLEN) and the samples were stored at − 20 °C until examination.

### Overlap extension PCR

**Fig. 1:**
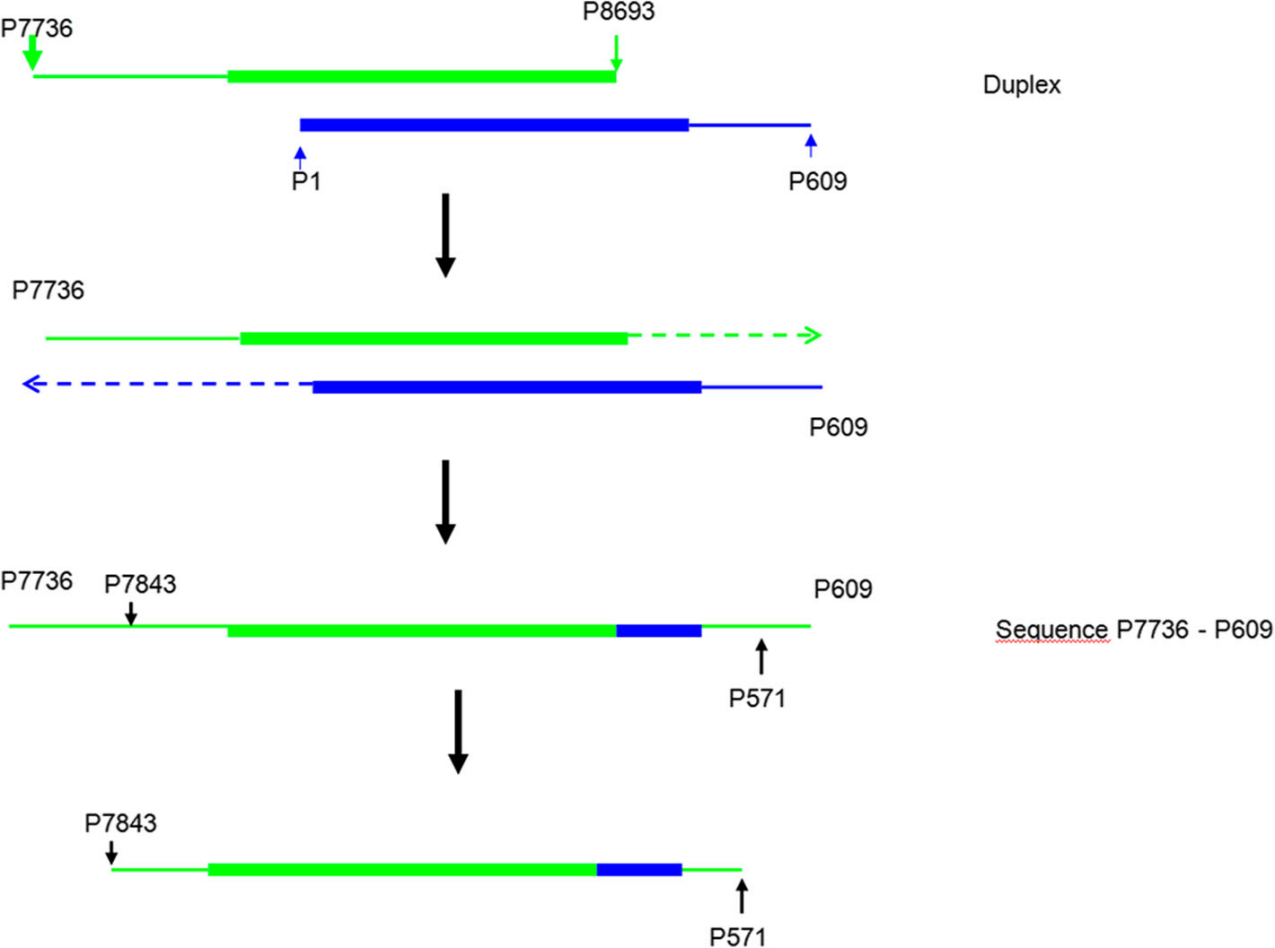
Scheme of the overlap extension PCR (OE-PCR) procedure. First, two overlapped LTR fragments are generated by regular PCR. Second, a DNA multimer is formed by OE-PCR without primers and with a prolonged extension time. The method significantly facilitate precise in-frame assembly of two LTR fragments

To amplify the BLV LTR sequences, an overlap extension PCR technique was developed. The general principle of the overlap extension PCR is shown in Fig. 1. This technique includes initial amplification of two fragments: 979 and 571 bp long. A 979 bp fragment was amplified using the following primers: P7736, 5′- TCGATACCCTCCTTGTGGACC-3′; P8693: 5’-TGTTTGCCGGTCTCTCCTGGCC-3′. The conditions of amplification were as follows: 500 ng of template DNA, 1 x KAPA Buffer, 0.2 mM dNTP_s_, 0.4 μM each primer, 2 mM MgCl_2_ and 0.5 U of KAPA Taq Polymerase. The amplification was run in Biometra T-Personal Thermocycler under following conditions: initial denaturation 95 °C 5 min, subsequent steps 95 °C 30 s, annealing 65 °C 45 s, extension 72 °C 1 min, 38 cycles total, final additional extension 72 °C 7 min, hold at 4 °C. The second fragment a 571 bp long was amplified using the following set of primers: P1: 5’-TGTATGAAAGATCATGCCGA-3′; P609: 5′- GACCCAAAATGCCGCCGAG-3′, at the same conditions except the annealing condition at 52 °C 45 s and a 40-cycle run for the amplified product.

Finally, both PCR fragments were used at equimolar proportions as template to generate final fragment 914 bp long using the following set of primers: P7843: 5’-ATTCTACCCCTAGGCGAGCC-3′ and P571: 5’-GTTAGGGTTCCGGGGTGATC-3′ and the same content of reaction mixture. Thermal conditions were as follows: initial denaturation 95 °C 5 min, subsequent steps 95 °C 35 s, annealing 64 °C 50 s, extension 72 °C 1 min 15 s, 38 cycles total, final additional extension 72 °C 9 min, hold at 4 °C. The sequences of all primers were designed using Primer 3 web-based software, found at http://www.bioinformatics.nl/cgi-bin/primer3plus/primer3plus.cgi. For all amplifications, the PCR products were visualized by 1.5% agarose gel electrophoresis with Simply Safe (EURx) staining (5 μl /100 ml) in 1X TAE buffer.

### Sequencing of full-length LTR

The PCR products were purified using a NucleoSpin Extract II Kit (Marcherey Nagel GmbH & Co) and subsequently, three independent PCR amplification products of each BLV isolate were sent to Genomed SA Company (Warsaw, Poland) for DNA sequencing in both directions, using a 3730xl DNA Analyzer (Applied Biosystems) and a Big Dye Terminator v3.1 Cycle Sequencing Kit. Raw sequence data were viewed and proofread in Geneious Pro version 5.5.9 (Biomatters Ltd) [27], the reads of each sample were aligned. A consensus sequence was determined for each BLV isolate. The data were deposited in the GenBank database under accession numbers included in Table 2.

**Table 2:** Mean of nucleotide distances for BLV LTR isolates. Values of the mean of estimates of evolutionary divergence over sequence pairs between groups

Origin/number		1	2	3	4	5	6	7	8	9	10
1	Poland (*n* = 52)										
2	Croatia (*n* = 2)	0.017									
3	Ukraine (n = 1)	0.012	0.017								
4	Moldova (n = 4)	0.024	0.029	0.021							
5	Russia (n = 5)	0.015	0.019	0.008	0.022						
6	Japan (*n* = 3)	0.016	0.013	0.013	0.023	0.016					
7	Myanmar (n = 2)	0.028	0.028	0.026	0.035	0.027	0.023				
8	USA (*n* = 2)	0.015	0.012	0.012	0.022	0.015	0.007	0.023			
9	South America(*n* = 8)	0.022	0.021	0.019	0.028	0.022	0.016	0.027	0.014		
10	Costa Rica (n = 2)	.0.029	0.028	0.026	0.037	0.028	0.024	0.032	0.023	0.028	

The number of base differences per site from averaging over all sequence pairs between groups is shown. The analysis involved 81 nucleotide sequences. All positions containing gaps and missing data were eliminated. There was a total of 526 positions analyzed in the final dataset. The number of isolates in each group (n) is listed

### Sequence data analysis

The sequences generated from 64 field isolates from Poland (52), Moldova (4), Russia (5), Croatia (2) and Ukraine (1) were aligned with 17 sequences of previously characterized BLV isolates from other geographical locations using ClustalW algorithm implemented in Geneious Pro 5.5.9 (Table 1). The multiple alignment was submitted to the Modeltest version 0.1.10 for the best model selection according to the Akaike information criterion (AIC). As a result, the HKY85 substitution model was applied in Geneious Pro to infer a phylogenetic tree according to Bayesian method. Genetic distance analysis between newly obtained sequences and average nucleotide substitution per site were calculated using the Jukes-Cantor model in MEGA version 5.2.2. Statistical analyses were performed using STATISTICA version 10 (StatSoft, Dell Software, USA).

To detect selection on noncoding DNA we used similar approach to Hahn et al. and Zhao et al. [22, 28]. A standard indication of selective pressure is the ratio of dN/dS, where dN/dS ~ 1 signifies neutral evolution or a balance in evolutionary pressure on that sequence, dN/dS < 1 indicates negative or purifying selection and a ratio > 1 indicates positive selection pressure [29, 30]. By analogy, we measured the ratio of the substitutions per site in regulatory sites (V_R_) to non-regulatory sites (V_NR_) in LTR region, with the same interpretation of results. To compare variability (number of substitutions per nucleotide per strain) in V_R_ versus V_NR_ sequences of LTRs representing 64 BLV isolates, the Friedman test was applied. The following regulatory elements of the LTR were analyzed: TRE1 (CRE1, E box 1), TRE2 (CRE2, E box 2), TRE3 (CRE3, E box 3), PU.1/Spi-B, κB, GRE, TATA Box, PAS, CAP site, USF, DAS (BoxA, BoxB, BoxC), Poly(A) and IRF. The remaining sequences within the LTR were considered as non-regulatory sequences. The level of variability between particular subregions of the LTR (U3-R-U5) was assessed using Wilcoxon matched pair test.

In further study, the consensus sequence was calculated based on the multiple alignment and it was used to determined nucleotide variations within each LTR in respect to known BLV genotypes (G1-G10). To analyze the transcription factor binding site (TFBS) modifications related to specific mutations, the multiTF server was used (http://multitf.dcode.org).

## Results

### Analysis of the sequence variation within BLV LTRs

Table 2 shows average genetic distances estimated for the 64 sequences obtained in this study, as well as 17 sequences from other studies (insert reference here), representing named reference sequences from other geographical locations. The level of divergence ranged between 0.012 to 0.037, when the two populations of sequences were analyzed. Genetic distances calculated for the 64 sequences varied between 0.008 and 0.029 and were comparable to those noted for reference sequences.

To identify the extent of genetic variations within the 64 LTR sequences, a consensus sequence was constructed (Additional file 1). Sequence variations were found amongst all BLV isolates. We found 97 (0.285%) point mutations distributed at 89 positions, along the 531 bp LTR amplicons of all 64 sequences. Among them, 46 were present in more than one isolate. The highest rate of variability was noted in U3 and U5 subregions (0.173 and 0.264, respectively), while the variability found in R subregion was relatively low (0.140). In fact, statistically significant differences were noted between the U5/R (*p*-value = 0.001155) and U3/R (*p*-value = 0.000002).

The most frequent changes were observed in regulatory elements of the U3 subregion and were characterized by substitution of G(− 133)A/C in the TRE2 of seven sequences, substitution T(− 65)C in the GRE seen in 28 sequences, and substitution of T(− 41)A, T(− 37)A and T(− 36)C in the TATA Box, noted in 4, 9 and 17 sequences, respectively.

The remaining variations were noted in DAS regulatory element of R subregion, and were characterized by substitution of A(+ 150)G found in 8 sequences, T(+ 161)C found in 3 sequences, and substitution of TC(+ 188/9)CT and T(+ 190)C recognized in 11 sequences.

Despite the high variability within the U5 subregion, no accumulation of specific mutations within regulatory elements was noted. Additionally, the LTR sequence analysis revealed the presence of insertions and deletions in LTR. Four deletions at position C(− 72)del of GRE, T(− 11)del of CAP site, TC(+ 188/9)del of BoxC and A(+ 320)del in non-regulatory site were observed. Two insertions at T (+ 191) ins and C (+ 192) ins were found in DAS and a non-regulatory site, respectively.

### Selection pressure in BLV LTR sequences

A substantial number of SNPs found in regulatory sequences of LTR suggested the possibility of positive selective pressure on BLV LTRs. To examine this hypothesis, the variability in regulatory (V_R_) versus non-regulatory (V_NR_) sequences was calculated and compared using Wilcoxon matched pair test. The respective results were shown in Table 3. Variability in regulatory sequences (V_R_) was lower than the variability within non-regulatory sequences (V_NR_) for both, U3 and U5 subregions. V_R_ versus V_NR_ was lower than 1 showing a high probability of negative selection (p< 0.05 for U3 and p< 0.0005 for U5). Conversely, V_R_ value for R subregion, was higher than the V_NR_ suggesting the existence of positive selection (p< 0.0001). For total LTR non-significant positive selection was predicted (*p* < 0.900).

**Table 3:** Evidence of selective pressure in regulatory sequences of U3, R, U5 regions and total BLV LTRs

	U3		R		U5		Total LTR	
	Mean	Variance	Mean	Variance	Mean	Variance	Mean	Variance
V_R_	0.009141	0.000068	0.012219	0.000220	0.005188	0.000410	0.010031	0.000048
V_NR_	0.013453	0.000167	0.00450	0.000031	0.017406	0.000270	0.009906	0.000054
V_R_/V_NR_	0.679443		**2.715278**		0.298025		**1.012618**	
*p*-value	< 0.05		< 0.0001		< 0.0005		< 0.900	

*V*_*R*_ Variability in regulatory sequences (regulatory elements)_,_ *V*_*NR*_ Variability in non-regulatory sequences, *V*_*R*_*/V*_*NR*_ The ratio was calculated to obtain evidence of selection; < 1 for negative, =1 for neutral and > 1 for positive selection. V_R_/V_NR_ values representing positive selection was bolded

### Phylogenetic analysis

**Fig. 2:**
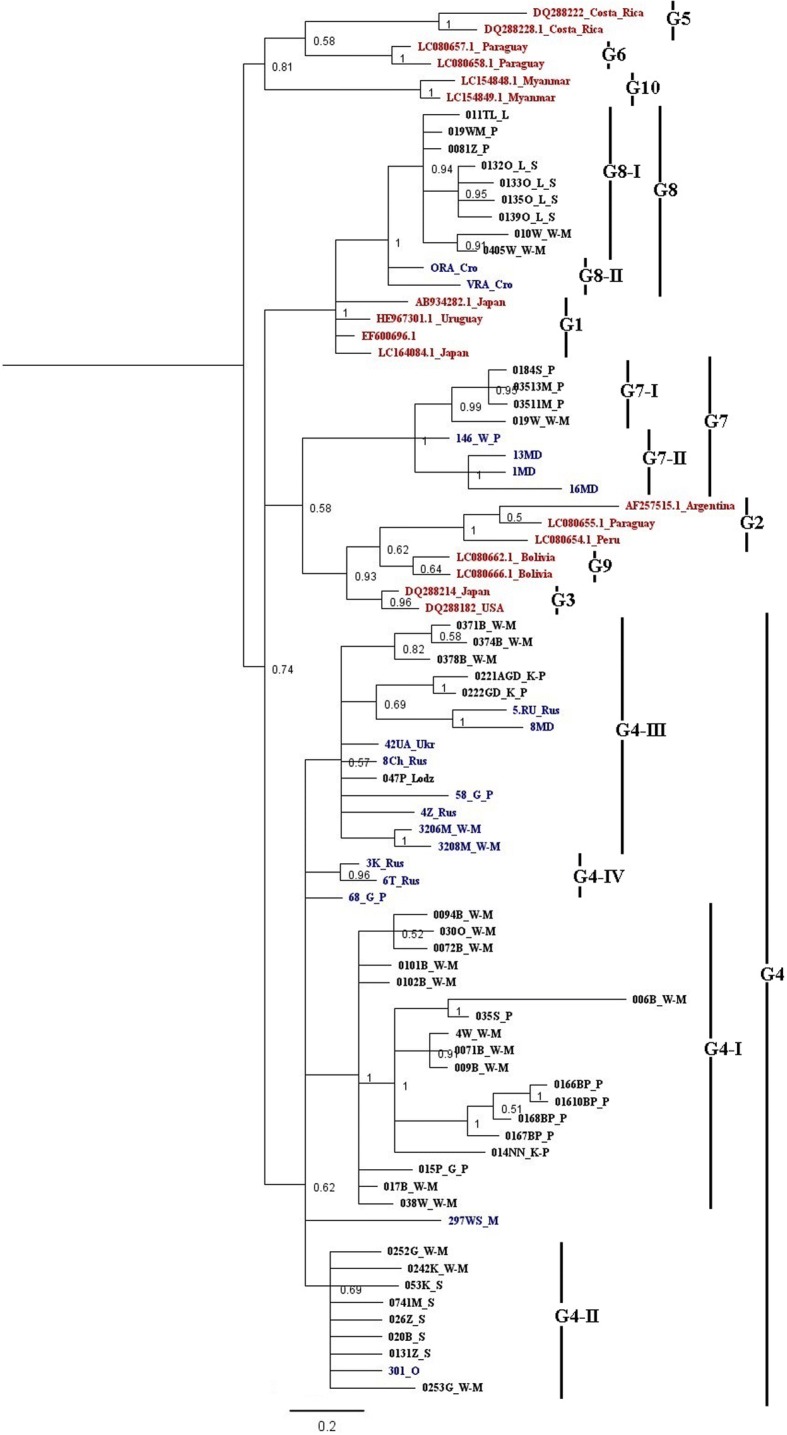
Phylogenetic relationship of BLV genotypes and subtypes. The relationships between sequence data obtained in this study between 2009 and 2012 (indicated by blue color, *n* = 17), selected from EBL monitoring programmes between 2013 and 2016 (indicated by black color, *n* = 47) and additional reference sequences in GenBank (dark red color, *n* = 17) were inferred by Bayesian analysis of LTR sequences, based on the HKY85 substitution model. Novel and known genotypes and/or subtypes found in this study are indicated at the right by vertical lines

Based on the multiple sequence alignment of 64 sequences and 17 reference sequences, a phylogenetic tree was constructed using the Bayesian method. The topology, with high posterior probabilities, indicated that all sequences were clearly classified into ten distinct groups (Fig. 2). This topology fully matched the phylogenetic tree which was constructed on the basis of a 444 bp fragment of the *env* gene, representing the same BLV isolates, with the presence of ten known BLV genotypes (G1-G10).

Analyzed sequences were segregated into three main groups, corresponding to the G4, G7 and G8 genotypes. Forty-five sequences representing Polish isolates and sequences from Moldova, Russia and Ukraine were clustered together within G4 genotype with a posterior probability of 0.62. Further, they were clearly separated into distinct subgroups, designated as G4-I, G4-II, G4-III and G4-IV. G4-III was cosmopolitan group including sequences from Poland, Ukraine, Russia and Moldova, while the remaining G4 subgroups represented only sequences from Poland.

The genotype G8, with high posterior probability score 1.00, grouped nine sequences from Poland and two sequences from Croatia, creating two separated subgroups G8-I and G8-II, respectively. All eight remaining sequences were found to create a G7 genotype with high posterior value 1.00. Five out them exclusively represented sequences from Poland forming a G7-I subgroup, while three sequences from Moldova were assigned to the G7-II subgroup.

### Selection pressure in genotypes and subtypes

The variability in V_R_ versus V_NR_ sequences was further calculated to estimate any selection pressure on LTR regulatory elements in individual genotypes and subgroups detected in this study. The respective results were shown in Table 4. For most of the phylogenetic groups, the LTR sequences were under negative or neutral selection. However, positive selection was estimated in the R region of the LTR sequences representing genotype G4 (*p* < 0.006039) and G8 (*p* < 0.000002). In particular, it was noted for the G4-III (*p* < 0.029411) cosmopolitan subgroup including Polish, Ukrainian, Moldavian and Russian sequences, as well as for G8-I (*p* < 0.011078) subgroup of Polish sequences. Positive selection was also detected in the U3 subregion (*p* < 0.000020) and total LTR (*p* < 0.000001) of G4-I subgroup which included Polish sequences.

**Table 4:** Evidence of selection on BLV total LTR in each genotype and subtype

	LTR phylogenetic groups	Sample size (no isolates)	*p*-value (V_R_ ≠ V_NR_)			
			U3	R	U5	Total LTR
Genotype	G4	45	> 0.05	0.006039 ^ȣ^	> 0.05	> 0.05
	G7	8	0.008158	> 0.05	0.000583	0.003496
	G8	11	0.000003	0.000002 ^ȣ^	0.007850	> 0.05
Subtype	G4-I	18	0.000020 ^ȣ^	> 0.05	0.040750	0.000001 ^ȣ^
	G4-II	9	> 0.05	> 0.05	0.000003	0.001179
	G4-III	14	0.000034	0.029411 ^ȣ^	> 0.05	> 0.05
	G4-IV	2	> 0.05	> 0.05	> 0.05	> 0.05
	G7-I	4	> 0.05	> 0.05	0.03342	0.044321
	G7-II	3	> 0.05	> 0.05	> 0.05	> 0.05
	G8-I	9	0.014542	0.011078 ^ȣ^	> 0.05	> 0.05
	G8-II	2	> 0.05	> 0.05	> 0.05	> 0.05

^ȣ^ - indicates *p*-value where V_R_ was significantly higher than V_NR_, positive selection; *p*-value not marked with an *ou* mean that V_R_ was significantly lower than V_NR_, negative selection; *p*-value > 0.05 mean not significant difference between V_NR_ and V_R_; the 68_G_P, 146_W_P and 297WS_M sequences were not classified to the subtypes giving 61 sequences to the subtype analysis

### Genotype-specific variability

Based on the analysis of the multiple sequence alignment of 64 and 102 BLV LTR sequences collected in GenBank database (Additional file 2) it was possible to identify 15 unique SNPs for each of known genotype (G1-G10) and 6 subgroup-specific SNPs, which are presented in Table 5. Overall, 21 genotype and subgroup-specific SNPs at 19 positions were found. Nine out of 21 SNPs identified were located in TRE1, TRE2, GRE/TRE3, TATA and DAS regulatory elements of the LTR. The remaining 12 were equally distributed among the non-regulatory sequences of the U3, R and U5 subregions.

**Table 5:**
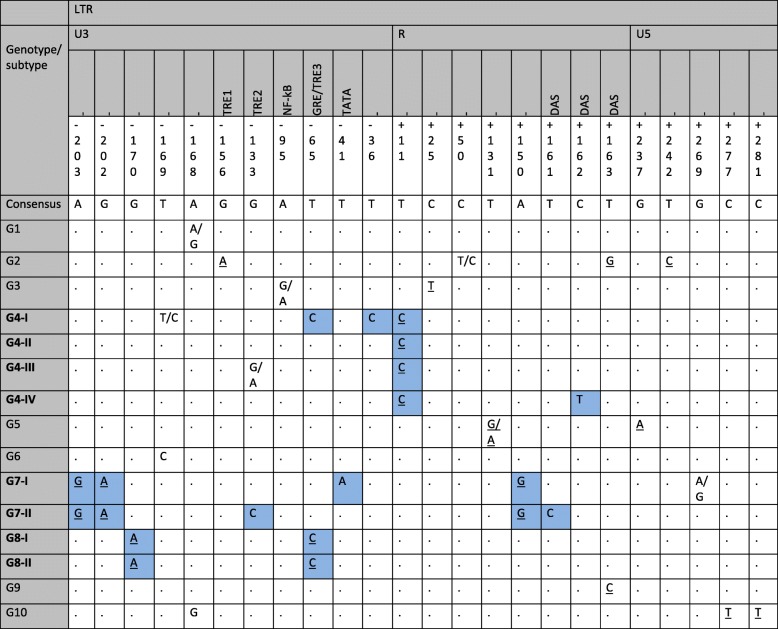
Unique LTR nucleotide differences which permit genotype/subtype classification

′Genotype/subtype′ column include pattern of the nucleotide sequence for each genotype and subtype, G1-G10 as well as G4-I, G4-II, G4-III, G4-IV, G7-I, G7-II, G8-I and G8-II, respectively. Nucleotides are represented by single letters and numbered from the beginning of 5′ to 3′ end of LTR. Blue squares represent unique SNPs for G4, G7 and G8 genotypes and/or subtypes. Underlined nucleotides represent specific SNPs for genotypes G2-G10

Here we report that genotype/subgroup-specific variations appeared to be geographically stratified throughout locations of the donor cattle. Some SNPs were found exclusively in isolates from certain geographic areas: e.g. T(− 41)A and T(− 36)C in only Polish isolates; G(− 133)C, T(+ 161)C for only Moldovan isolates; C(+ 162)T for Russian isolates), while some were found in isolates from multiple areas (e.g G(− 170)A and T(− 65)C for Polish and Croatian isolates; A(− 203)G, G(− 202)A, A(+ 150)G for Polish and Moldavian isolates; T(+ 11)C for Polish, Ukrainian, Moldavian and Russian isolates.

The detection of SNPs in regulatory elements suggested that there may be differential BLV expression in host infected with particular genotypes and/or subgroups. Five out of 9 SNPs identified in regulatory elements corresponded with G4, G7 and G8 genotypes. Another six out of 12 SNPs typical for these genotypes were identified in non-regulatory binding sites of the LTR.

To analyze transcription factor binding site (TFBS) modifications related to genotype and subgroup-specific SNPs for G4, G7 and G8 genotypes we used multiTF software. The data were evaluated for potential cellular transcription factors binding sites using an in silico analysis, in conjunction with SNPs that possibly abrogated existing TFBS or created a new TFBS (Table 6).

**Table 6:** Potential binding sites for the cellular transcription factors that could be implicated in LTR activity. The analyzed mutation position relate to genotype and/or subtype specific SNPs for G4, G7 and G8 genotypes

Mutation position	Change	Previously described regulatory elements	TFBS by multiTF	TFBS change		Genotype/subtype
				Creation	Abrogation	
−203	A → G	None	TFIID, GATA-1, Pit-1a	AP-1	TFIID, GATA-1	G7-I, II
−202	G → A	None	TFIID, GATA-1, Pit-1a	AP-1	TFIID, GATA-1	G7-I, II
−170	G → A	None	Sp1	PR	–	G8-I, II
−133	G → C	TREx2	XBP-3, GATA-1	CREM-tau2, AP-1	XBP-3, GATA-1	G7-II
−65	T → C	GRE/TREx3	CIIIB1	GATA-1	CIIIB1	G4-I, G8-I, II
−41	T → A	TATA Box	GATA-1, TMF, Pit-1a, TFIID, Sp1, PR	NF-E2, RAR-gamma2	GATA-1, TMF	G7-I
−36	T → C	None	TBP, AFP1, Pit-1a, GATA-1, TFIID	AP-1, ACF	TBP, AFP1	G4-I
+ 11	T → C	None	Sp1, PR	AP-1	–	G4-I, II, III, IV
+ 150	A → G	None	GATA-1	–	–	G7-I, II
+ 161	T → C	DAS	PR	–	–	G7-II
+ 162	C → T	DAS	PR	Tf-LF1	–	G4-IV

*TFIID* Transcription factor II D, *GATA-1* Erythroid transcription factor, *Pit-1a* Growth hormone factor 1, *AP-1* Activator protein 1, *Sp1* Specificity protein 1, *PR* Progesterone, *XBP-3* X-box binding protein 1, *CREM-tau2* Transcription factor cAMP-response element modulator protein, *CIIIB1* Nuclear factor, *TMF* TATA element modulatory factor, *NFE2* TF interacting with CREB-binding protein, *RAR-gamma2* Retinoic acid receptor gamma, control peptide, *TBP* TATA-binding protein, *AFP1* Hepatoma nuclear factor that binds to the alpha-fetoprotein enhancer and promoter, *ACF* ATP-dependent chromatin-assembly factor, *Tf-LF1* Liver-specific transcription factor

Five SNPs, identified in TREx2, GRE/TREx3, TATA Box, and DAS were contained XBP-3, GATA-1, CIIIB1, TMF, Pit-1a, TFIID, Sp1 and PR binding predicted sites as determined by in silico analysis. Variations disrupted putative sites for XBP-3, GATA-1, CIIIB1 and TMF, as well as possibly created new CREM-tau2, AP-1, GATA-1, NF-E2, RAR-gamma2 binding sites.

Six other SNPs that were not identified in common regulatory binding motifs were present in TFIID, GATA-1, Pit-1a, Sp1, TBP, AFP1 and PR binding putative sites determined by in silico analysis. Variations disrupted the predicted sites for TFIID, GATA-1, TBP and AFP1, and may have created new AP-1, PR, ACF binding putative sites. Future studies are required to assess the functional capacity of BLV promoters from distinct genotypes with divergent TF binding sites.

## Discussion

In this study we determined the nucleotide sequences for LTRs of BLV proviruses isolated from naturally-infected cattle from Poland, Moldova, Ukraine, Russia and Croatia. The 64 sequences analyzed proved to be 96.3–98.2% identical to the other BLV sequences isolated from cattle living in Japan, Myanmar, North, Central and South America. This data revealed that the genetic distances between BLV LTR sequences were very low among infected cattle and were comparable to that observed for coding sequences within the BLV genome. It was further shown that the homology of the *env* gene was 94.5–97.7% amongst BLV isolates representative of the ten genotypes.

Comparison the 64 LTR sequences with the previously described sequences from American and Asian countries reveals numerous SNPs which conferred differences throughout most of the length of the LTR. We propose that some of these differences may account for noted variability in viral transcription and replication. Expression of BLV is dependent on three imperfect 21 bp repeats (TRE), which are critical elements for both basal expression and for transactivation by the virally-encoded Tax protein. These TRE motifs contain imperfectly conserved cyclic AMP-responsive elements (CRE), specifically AGACGTCA, TGACGGCA, and TGACCTCA sites, that repress transcriptional activation by cellular CREB/ATF factors. This strategy avoids TRE site recognition during a host immune response [10]. We noted TRE variations which were located outside, G(− 133)A/C, T(− 65)C, and within the corresponding CRE octanucleotide, C(− 53)A, which created an imperfect CRE (TGACCTCA). Besides, each TRE contains an E-box sequence (CAGCTG, CAGCTG and CACCTG) which overlaps the CRE motif. E boxes are can exert a negative cooperative effect on the activity of the BLV promoter through the CREs, most likely by sterically inhibiting the binding of CREB/ATF to these sites [31]. We found E box variations, T(− 147)C, C(− 123)G, C(− 48)T and T(− 47)C, which may be involved in potentiating the basal activation of LTR-directed gene expression by CREB/ATF family proteins. Other variations observed include T(− 41)A, T(− 37)A and T(− 36)C substitution in TATA Box, which is a core promoter element that binds the cellular factor TATA*-*binding protein (TBP). TBP binds other cellular factors, as well as RNA polymerase II, to initiate transcription from the DNA template. The site-directed mutants TA(− 41 to − 40)AT and TT(− 37 to − 38)TTT of the TATA Box were shown to have significantly stronger responsiveness to Tax protein in comparison to the wild type LTR [32].

The numerous DAS variations, located downstream to the transcription initiation site, C(+ 134)T and A(+ 150)G in BoxA, C(+ 166)T in BoxB, TC(+ 188/9)CT and T(+ 190)C in BoxC and mutations outside of the boxes in DAS G(+ 156)A, T(+ 161)C; C(+ 162)T; A(+ 180)G, T(+ 182)C, C(+ 183)T and T(+ 184)C observed in this study might also introduce discrepancies in promoter activity. Specifically, these were included in three conserved boxes, which were found to be identical with HTLV-1 DRE 1, that is essential for up regulation of the HTLV-1 promoter [12, 33]. Kashanchi et al. revealed that the 45 nt sequence DRE 1, located at the boundary of the R/U5 region of the LTR, is required for basal HTLV-1 transcription and plays an important role in the initiation and maintenance of virus replication [33]. There were also observed T(+ 256)C, T(+ 259)C/G and C(+ 264)T changes in IRF binding site of the U5 region, which may also play role in initiation of virus replication [13].

Despite the overall differences described above, the sequences of several regulatory elements of the LTR were conserved. These included the TRE1, CRE2, NF-κB-like motif and PU.1/Spi-B binding site of U3. Another interesting feature of BLV LTRs is the presence of a conserved kappa B (κB) site. Constitutive expression of NF-κB in B cells can induce low level of transcription during BLV infection, which can be up-regulated following immunological activation and thus initiate a positive feedback loop involving the Tax protein [13]. Two SNPs, T(− 104)C and T(− 84)C, were located within κB site and PU.1/Spi-B binding site, respectively. Notably, Xiao et al. revealed that TC(− 83 to − 84)CT substitution in the PU.1/Spi-B binding site increased BLV promoter function fivefold [32].

In our work, we investigated possible natural selection over the entire BLV LTR sequence, as well as particular regions of the LTR (U3, R and U5), from 64 new BLV isolates and found evidence of negative selection for most of the regulatory elements. These results are consistent with the observations of Zhao et al., who showed significantly higher variability of non-regulatory than regulatory sequences in LTRs based on the analysis of 52 BLV isolates from USA, Japan and Costa Rica [22]. Nevertheless, in contrast to their findings of no evidence for positive selection for all LTR regions, we found evidence that regulatory sequences within R region were under positive selection, specifically the CAP site, USF, DAS: BoxA, BoxB, BoxC and Poly (A) sites. The essential difference in selection between these studies could be dictated by including the DAS element in this study, in contrast to Zhao and coworkers, who excluded it from their analysis. DAS is a 64 bp long sequence present at the 3′ end of the R region, which contributes to the enhancement of viral gene expression [12]. Interestingly, BLV DAS region harbors both progesterone and glucocorticoid binding sites, indicating a putative role in RNA synthesis. Asin et al. demonstrated that in HIV-1-infected cells, progesterone and estradiol regulate HIV-1 replication most likely by directly altering HIV-1 transcriptional activation and in the absence of the viral regulatory protein Tat [34]. Our analysis revealed that, unlike viral essential *cis*-acting promoter control sequences contained within U3 region (TRE1, CRE2, κB and PU_Box), R region can undergo continuous substitutions due to host selection pressure. This sequence variation of target transcription factor binding sites in R region may reduce the binding sites recognition and potentially be detrimental to the efficacy of transcription factors on the BLV LTR and/or hormone responsiveness. Since glucocorticoid (GR) and progesterone (PR) receptors are known to drive the activity of the HIV-1 and select endogenous retroviruses, we believe they may likely also modulate the activity of BLV 5′ LTR [8, 34, 35].

Another finding emerging from the in silico analysis is the possibility that many additional cellular transcription factors could contribute to the regulation of BLV transcription. We found that genotype and subgroup-specific SNPs can lead to creation or abrogation several transcription factors in the LTR. The putative binding sites for transcription factors, unique for particular genotypes/subgroups, were observed notably for AP-1, PR, CREM-tau2, NF-E2, RAR-gamma2, GATA-1 and ACF. We postulate that these observed variations can lead to genotype/subtype- specific differences in viral gene expression.

In support of this concept, Jeeninga et al. revealed that the change from κB to GABP binding site in the LTR of HIV-1 subtype E did not lead to loss of promoter function; instead, a gain of GABP binding to this mutated κB binding site enhanced Tat-mediated gene expression and improved virus replication in select cell lines [36]. We found that the SNP G(− 133)C, specific for G7-II, located in TREx2 regulatory element, was found to introduce CREM-tau2 and AP-1 potentially leading to abrogation of the XBP-3 and GATA-1 binding putative sites. Interestingly, CREM isoforms are transcription factors belonging to the CREB/CREM/ATF family, which are known to cooperate with activator protein 1 (AP-1), were upregulated by Tax protein [37]. AP-1 transcription factor family include FOS, JUN, MAF, and ATF proteins, which are significantly upregulated in the presence of Tax [37, 38]. This AP-1 can form different AP-1 dimers, which control transcriptional activation or suppression of many genes.

The SNP T(− 41)A in the TATA Box, typical for the G7-I subtype, harbors a shift from an GATA-1/TMF to NF-E2/RAR-gamma2 binding putative sites. Interestingly, transcription factor NF-E2 is known to bind with a specific DNA sequence in order to modulate transcription by RNA polymerase II. These newly determined, in silico predicted TFBS can be useful for further experiments directed at defining the physical interactions between viral and host regulatory proteins, and may help define the biochemical processes of basal transcription and Tax transactivation in B cells.

## Conclusion

Our study represents the largest study of the LTR genetic variability of BLV field isolates from Eastern part of Europe. The SNP screening showed variations modifying LTR regulatory sequences, as well as altering existing TFBS. Different sets of mutations for LTRs from different genotypes and subtypes suggests that multiple, geographically localized changes, may be involved in viral adaptation to recent selective pressures. These adaptations may underlie differential clinical outcomes of BLV infection, with potential implications for agricultural practice and economy [39–41].

## Additional file

Additional file 1: Alignment of 60 representative full-length BLV LTR sequences. The labeled rectangles in the upper part of figure refer to the regulatory elements of LTR. Dots indicate identity with consensus sequence, generated based on the 81 sequences analyzed in this study. Abbreviations are included in the attached list of abbreviations. Classification of the sequences according to the genotypes/subtypes is shown in the left part of the figure. (ZIP 281 kb)

Additional file 2: LTR sequences from a total of 102 BLV field isolates used in this study, with GenBank accession numbers and geographic origin. (XLSX 14 kb)

## Abbreviations

ACF: ATP-dependent chromatin-assembly factor
AFP1: Hepatoma nuclear factor that binds to the alpha-fetoprotein enhancer
AGID: Agar gel immunodiffusion
AP-1: Activator protein 1
ATF-1: Activating transcription factor-1
BLV: Bovine leukemia virus
bZip domain: Basic leucine zipper domain
CIIIB1: Nuclear factor
CRE: Cyclic AMP-responsive elements
CREB: Cyclic AMP response element binding protein
CREM-tau2: Transcription factor cAMP-response element modulator protein
DAS: Downstream activator sequence
EBL: Enzootic bovine leukosis
ELISA: Enzyme-linked immunosorbent assay
ETS: E26 transformation-specific (ETS) transcription factors
GATA-1: Erythroid transcription factor
GRE: Glucocorticoid response element
HBZ: HTLV-1 bZIP factor
HIV: Human immunodeficiency virus
HTLV: Human T-cell lymphotropic virus
IRF: Interferon regulatory factor
LTR: Long terminal repeat
NFE2: TF interacting with CREB-binding protein
NF-κB: Nuclear factor kB
PAS: Poly (A) signal sequence
PBLs: Peripheral blood leukocytes
Pit-1a: Growth hormone factor 1
PR: Progesterone
PU.1/Spi-B: Spi-B transcription factor
RAR-gamma2: Retinoic acid receptor gamma
SNP: Single nucleotide polymorphisms
Sp1: Specificity protein 1
TBP: TATA-binding protein
TFBS: Transcription factor binding site
TFIID: Transcription Factor II D
Tf-LF1: Liver-specific transcription factor
TMF: TATA element modulatory factor
TNFα: Tumor necrosis factor α
TRE: Tax-responsive element
USF: Upstream stimulatory factor
VNR: Non-regulatory sequences
VR: Regulatory sequences
XBP-3: X-box binding protein 1
κB: DNA binding site for nuclear factor kB
