# Supplementary figures and images for: Bovine leukemia virus long terminal repeat variability: identification of single nucleotide polymorphisms in regulatory sequences
Source: Virol J. 2018 Oct 25;15:165. doi: 10.1186/s12985-018-1062-z (PMC6202831; doi:10.1186/s12985-018-1062-z)

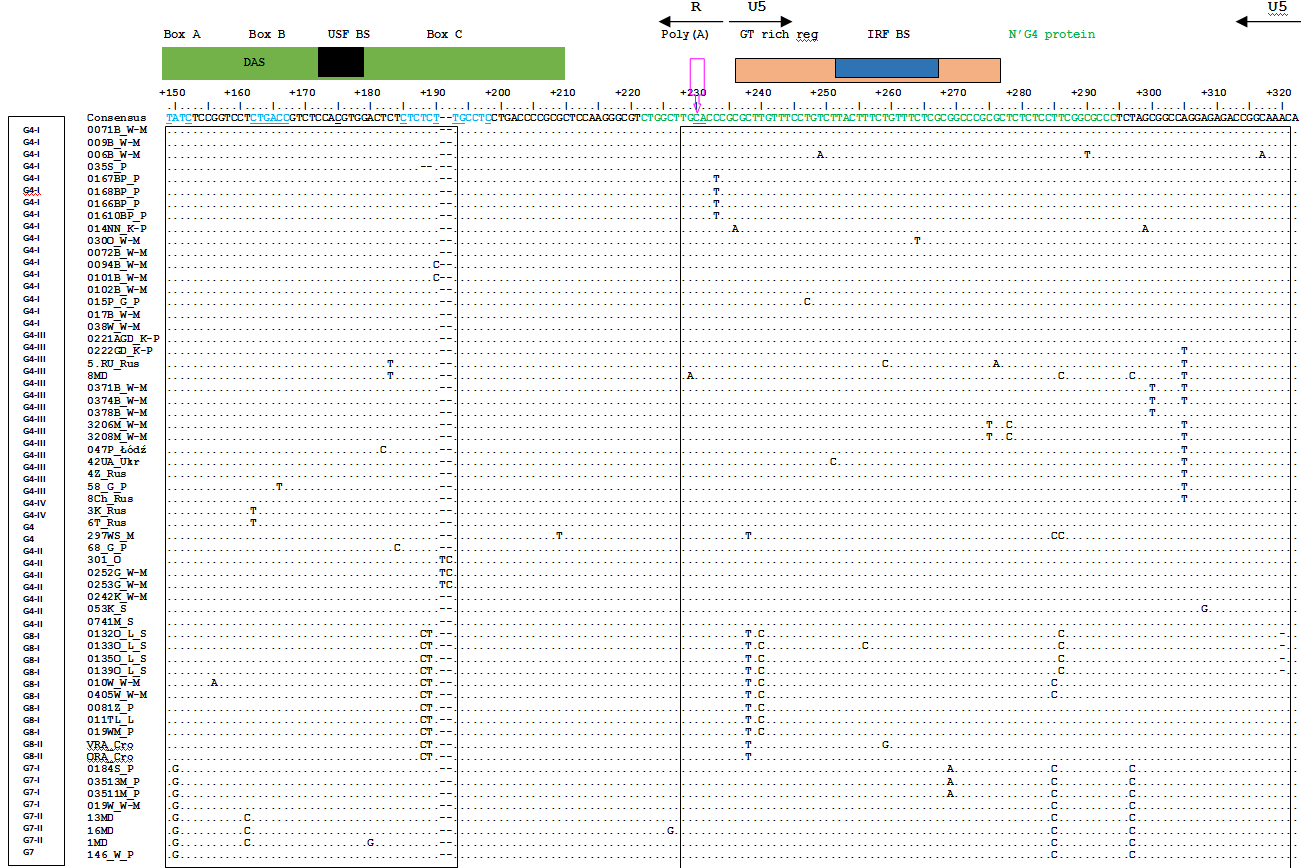

Supplement: Supplementary file 1 — Alignment of 60 representative full-length BLV LTR sequences. The labeled rectangles in the upper part of figure refer to the regulatory elements of LTR. Dots indicate identity with consensus sequence, generated based on the 81 sequences analyzed in this study. Abbreviations are included in the attached list of abbreviations. Classification of the sequences according to the genotypes/subtypes is shown in the left part of the figure. (ZIP 281 kb) [file 12985_2018_1062_MOESM1_ESM.zip › Figure 2_part III_ continue.tiff]

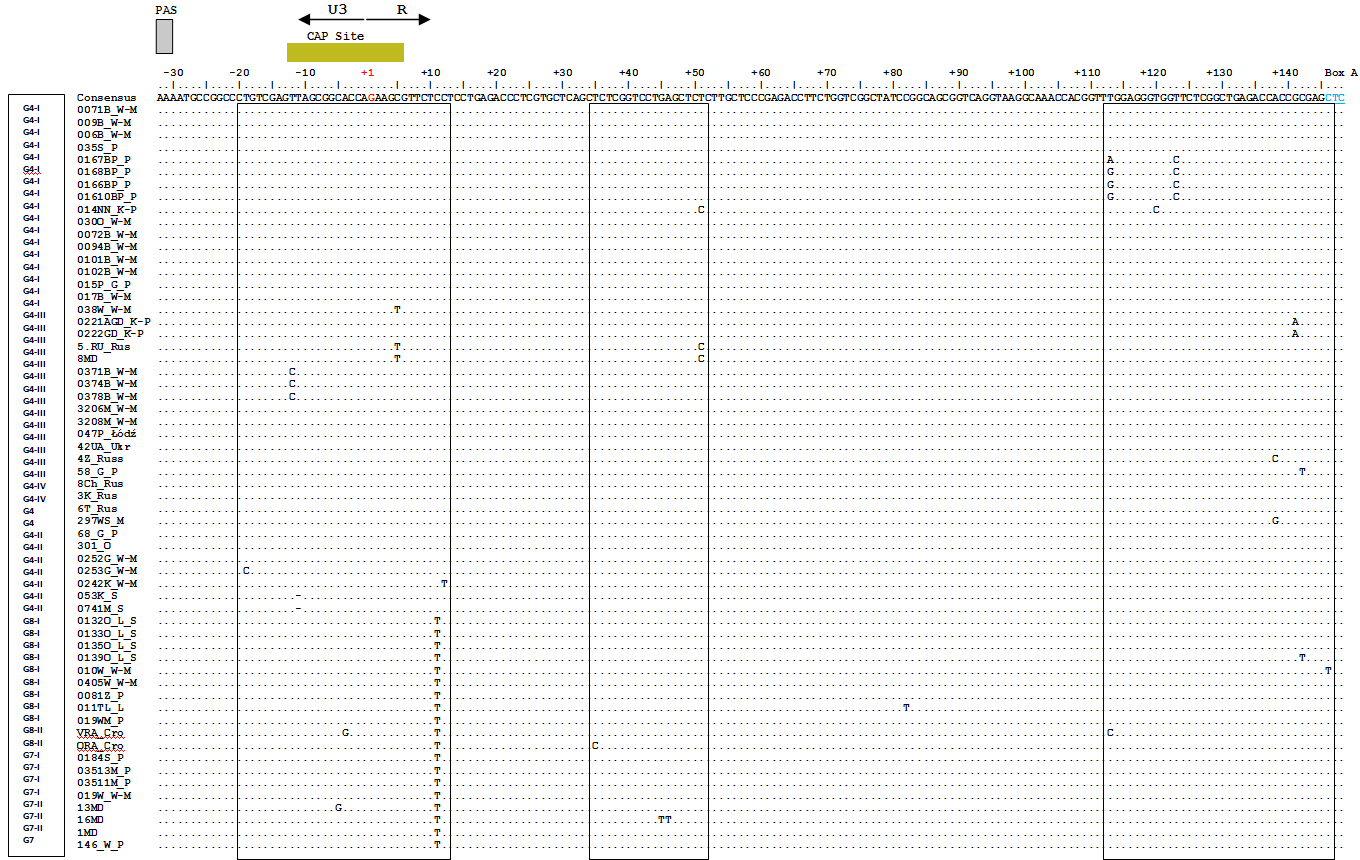

Supplement: Supplementary file 1 — Alignment of 60 representative full-length BLV LTR sequences. The labeled rectangles in the upper part of figure refer to the regulatory elements of LTR. Dots indicate identity with consensus sequence, generated based on the 81 sequences analyzed in this study. Abbreviations are included in the attached list of abbreviations. Classification of the sequences according to the genotypes/subtypes is shown in the left part of the figure. (ZIP 281 kb) [file 12985_2018_1062_MOESM1_ESM.zip › Figure 2_part II_continue.tiff]

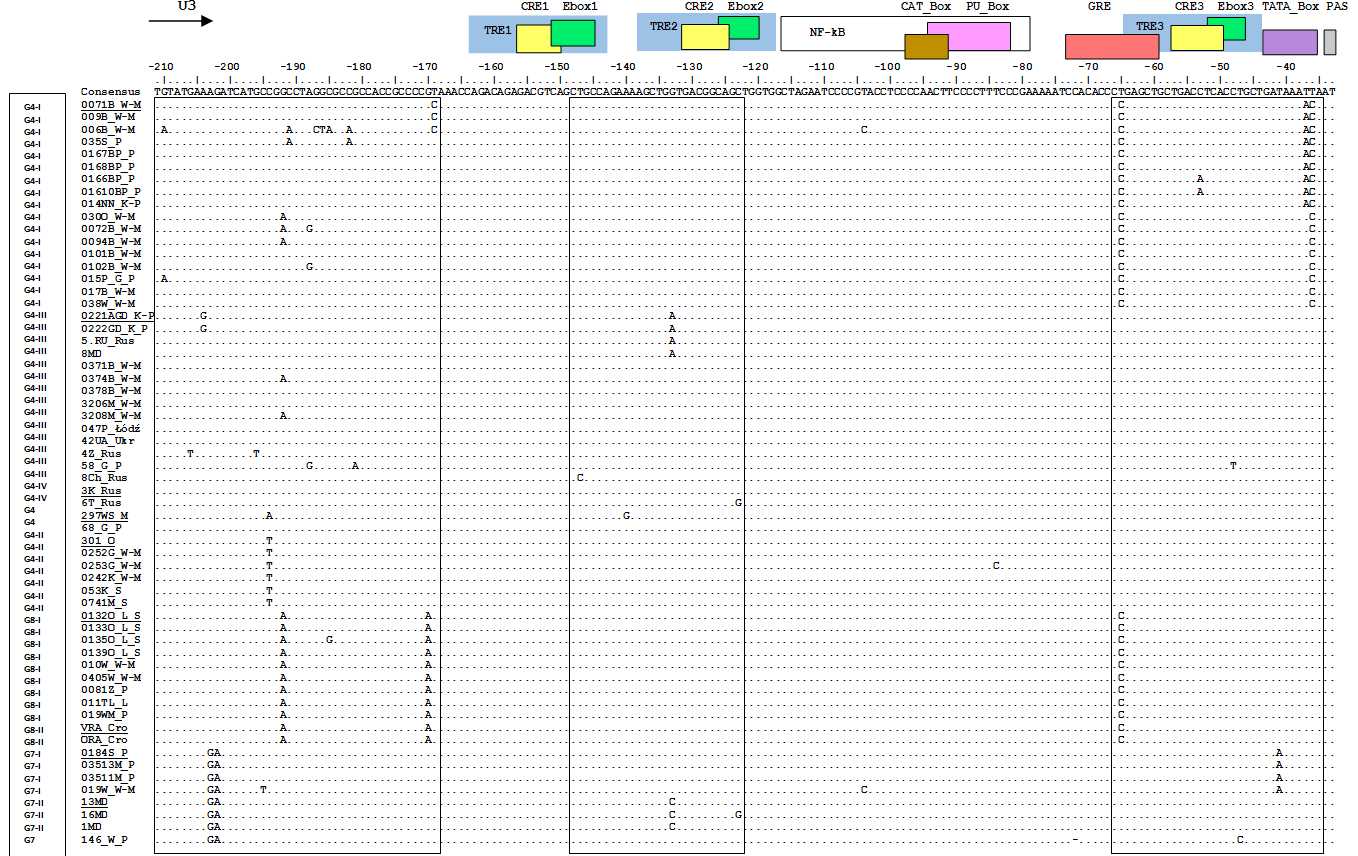

Supplement: Supplementary file 1 — Alignment of 60 representative full-length BLV LTR sequences. The labeled rectangles in the upper part of figure refer to the regulatory elements of LTR. Dots indicate identity with consensus sequence, generated based on the 81 sequences analyzed in this study. Abbreviations are included in the attached list of abbreviations. Classification of the sequences according to the genotypes/subtypes is shown in the left part of the figure. (ZIP 281 kb) [file 12985_2018_1062_MOESM1_ESM.zip › Figure 2_part I.tiff]
